# Supplementary material for: Preconfigured cortico-thalamic neural dynamics constrain movement-associated thalamic activity
Source: Nat Commun. 2024 Nov 24;15:10185. doi: 10.1038/s41467-024-54742-9 (PMC11586408; doi:10.1038/s41467-024-54742-9)
Supplement: Supplementary file 1 — Supplementary Information [file 41467_2024_54742_MOESM1_ESM.pdf]

Preconfigured cortico-thalamic neural dynamics constrain movement-associated thalamic activity

Perla González-Pereyra[1], Oswaldo Sánchez-Lobato[1], Mario G. Martínez-Montalvo[1], Diana I. Ortega-Romero[1], Claudia I. Pérez-Díaz[1], Hugo Merchant[1], Luis A. Tellez[2], Pavel E. Rueda-Orozco[1]*.

[1] Departamento de Neurobiología del Desarrollo y Neurofisiología, Instituto de Neurobiología, UNAM
[2] Departamento de Neurobiología Conductual y Cognitiva, Instituto de Neurobiología, UNAM

*Correspondence: Dr. Pavel E. Rueda-Orozco; e-mail: pavel.rueda@gmail.com; ruedap@unam.mx; Instituto de Neurobiología, UNAM Campus Juriquilla. Boulevard Juriquilla No. 3001, Querétaro, 76230, México.

Supplementary Figures

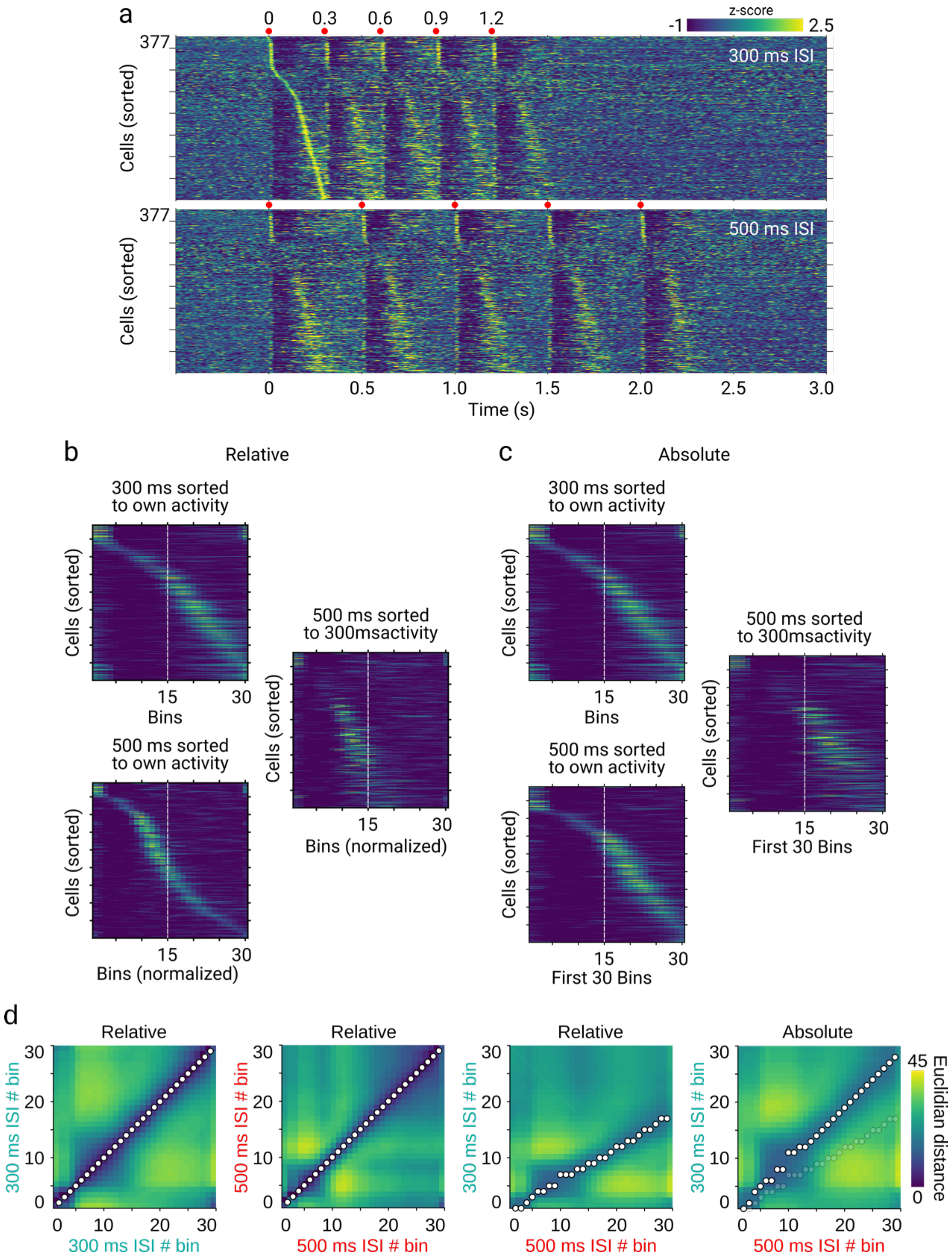

Supplementary Figure 1. Population temporal dynamics in VL/VM. a) Average firing rates (z-scored) evoked by M1 stimulation for cells recorded under two conditions: 300 ms (top) and 500 ms (bottom) ISI. Cells in both matrices were sorted according to the moment of their highest firing rate between the first and second stimulus of the 300 ms ISI train. Each stimulus of the train is indicated above each panel (red dots). Thirty-bin matrices depicting the average firing rates for the five stimuli of the train for relative (**b**) and absolute (**c**) comparisons. Cell sorting is indicated above each panel. For

500 ms relative matrices, neural activity was adjusted to 30 bins. For 500 ms absolute matrices, neural activity corresponds to the first 300 ms after the onset of stimulation. White dashed lines are depicted as a visual reference. d) Euclidean distance matrices comparing 300 ms and 500 ms dynamics in the relative (time normalized) or absolute sorted matrices displayed in b and c, respectively. The minimum distance per bin is depicted with circles. For the 300/500 ms absolute comparison, minimum distances for the relative comparison are also displayed in shaded circles as a visual reference.

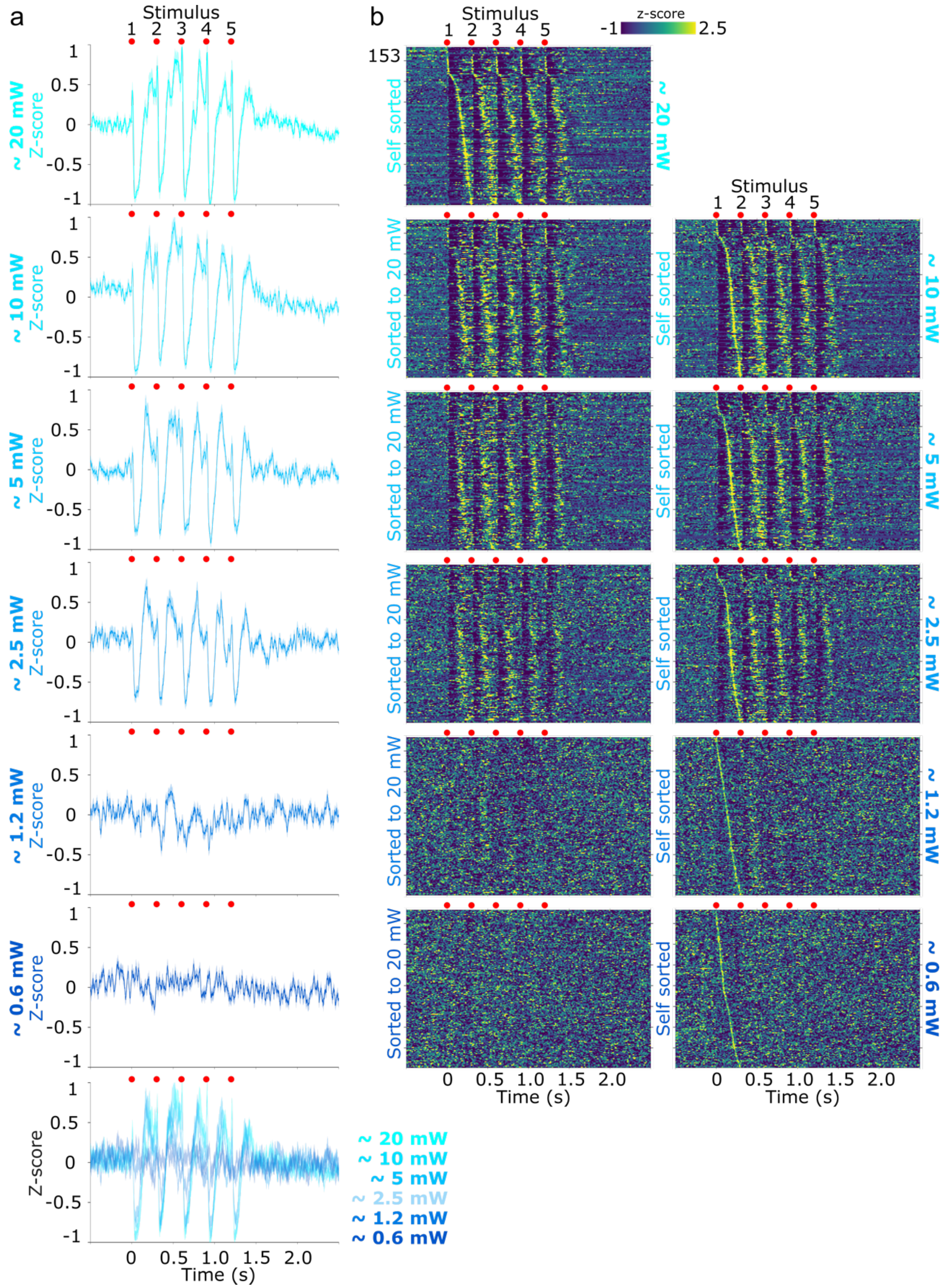

Supplementary Figure 2. M1-evoked response patterns at different stimulation intensities in awake conditions. a) Full train-averaged population response aligned to the first stimulus of the train for six light intensity stimulations (color code indicated on the Y axis) in naïve awake animals (same color code for the rest of the figure). For visual comparison, the bottom panel merged all conditions. **b)** Average firing rates (z-scored) evoked by M1 stimulation for the same cells recorded under the six light intensities. In the left column, cells were sorted according to the moment of their highest firing rate between the first and second stimulus of the train for the 20 mW stimulation. In the right column cells

were sorted according to the moment of their highest firing rate between the first and second stimulus of the train for each intensity (self-sorted). Fifty 300 ms ISI trains were given for each light intensity. Light intensities were provided in descending order starting at 20 mW.

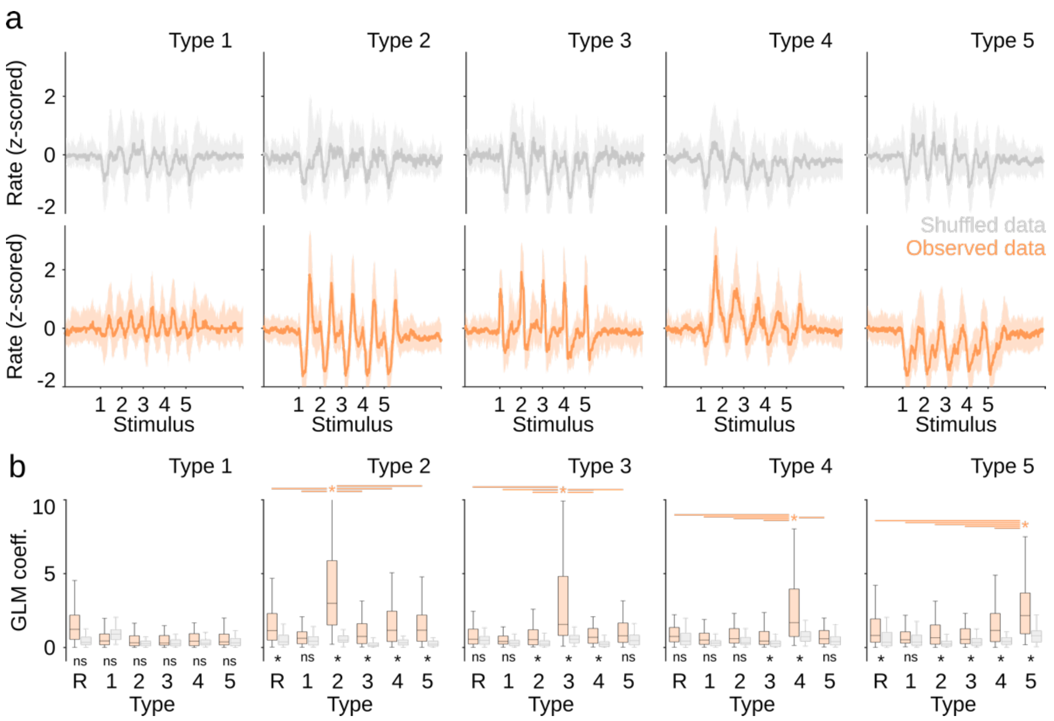

Supplementary Figure 3. Generalized linear model analysis for M1-evoked response patterns. a) Full train-averaged population M1-evoked patterns for cells classified as part of a specific pattern based on maximum generalized linear model (GLM) regression coefficients and from surrogate data (gray traces, upper row) and observed data (orange traces, lower row). **b**) GLM regression coefficients obtained for each group of neurons belonging to each pattern (orange boxplots) and surrogate data (gray boxplots). Boxplots indicated by number show the pattern type, while boxplots indicated by R represent the coefficients for a regressor generated with a randomized function. Boxplots indicate median and 75th and 25th percentiles. Statistical differences between coefficients generated with observed data between different pattern types are indicated by orange asterisks and orange lines joining specific comparisons in the upper region of each panel. Statistical differences between each pattern type and its corresponding surrogate data are shown by black asterisks at the bottom of each panel (Bonferroni post hoc test, * $P < 0.05$). Kruskal-Wallis values for each panel: Type 1, degrees of freedom *[df]* $= 11$, $X^2 = 151.5$, $p < 0.001$; Type 2, $df = 11$, $X^2 = 282.16$, $p < 0.001$; Type 3, $df = 11$; $X^2 = 142.24$; $p < 0.001$; Type 4, $df = 11$; $X^2 = 165.67$; $p < 0.001$; Type 5, $df = 11$; $X^2 = 260.27$; $p < 0.001$.

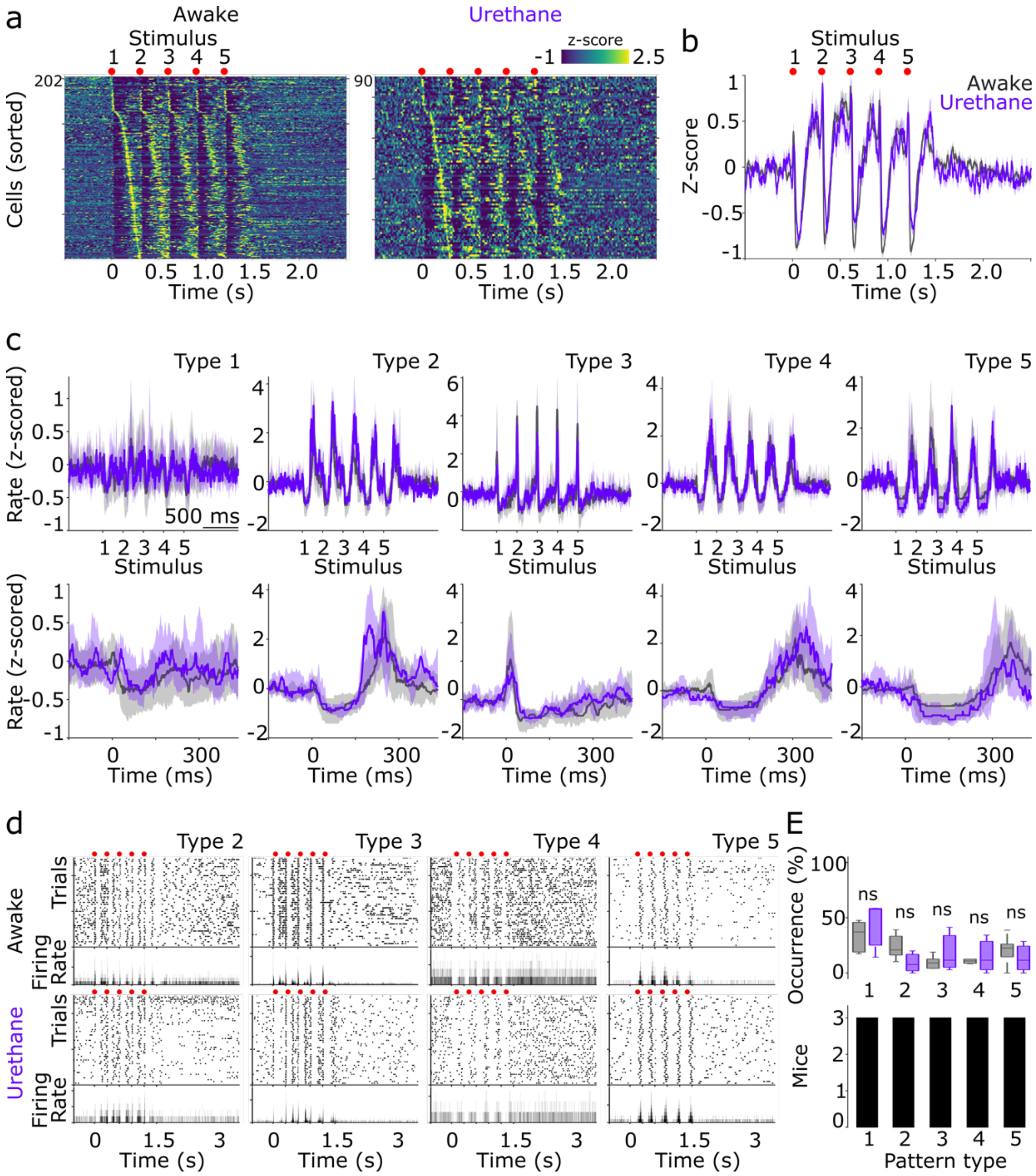

Supplementary Figure 4. M1-evoked response patterns in urethane anesthetized animals. a) Average firing rates (z-scored) evoked by M1 stimulation for cells recorded under two conditions: 300 ms awake (left) and 300 ms anesthetized (right) inter-stimulus intervals (ISI). Cells were sorted according to the moment of their highest firing rate between the first and second stimulus of the train. Each stimulus of the train is indicated above each panel (red dots; same for the rest of the panels). **b**) Full train-averaged population response aligned to the first stimulus of the train for the awake (gray traces) and anesthetized (purple traces) conditions (same color code for the rest of the figure). **c)** Average M1-evoked patterns for cells classified as part of specific pattern clusters for the awake and anesthetized conditions. Solid lines and shaded areas represent the median and the 25th and 75th percentiles, respectively. **d**) Representative spike rasters and their corresponding average peri-event histograms for eight different neurons belonging to specific pattern clusters recorded in awake (top row) and anesthetized (bottom row) conditions. Activity was aligned to the first stimulus of the train. **E**) Percentage of cells belonging to each pattern cluster displayed in A (upper panel). Number of animals that presented each pattern (lower panel). Boxplots indicate median and 75th and 25th percentiles. Statistical comparisons were performed by applying Kruskal-Wallis.

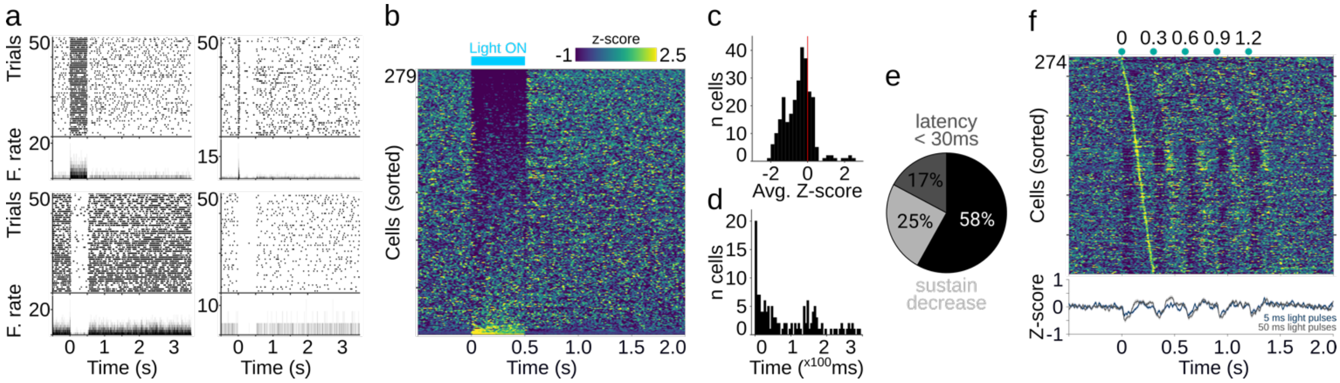

Supplementary Figure 5. Activation of cortical PT neurons. a) Representative spike rasters and their corresponding average peri-event histograms for four different neurons recorded in M1 with different responses to 500 ms light pulses delivered in the same area. **b**) M1 average firing rates (z-scored) evoked by 500 ms stimulation pulses. Cells were aligned to the onset of light stimulation and sorted according to maximum averaged firing rate during the stimulation period (indicated by blue bar on top of the panel). **c**) Histogram of averaged z-scored responses to during light stimulation. **d**) Response latencies for cells that increased their firing rate in response to the light stimulation. **e**) Percentage of cells that presented response latencies shorter than 30 ms or that presented a sustained decrease in activity (< 1 z-score averaged during 500ms) during light stimulation. **f**) Average firing rates (z-scored) evoked by light stimulation for cells recorded under the 300 ms inter-stimulus interval protocol (upper panel). Cells were sorted according to the moment of their highest firing rate between the first and second stimulus of the train. Each stimulus of the train is indicated above the panel (green dots). For comparison purposes the bottom panel depicts the full train-averaged population response aligned to the first stimulus of the train for the 5 ms (blue trace, from Fig. 3c) and 50 ms (gray trace) light pulse conditions.

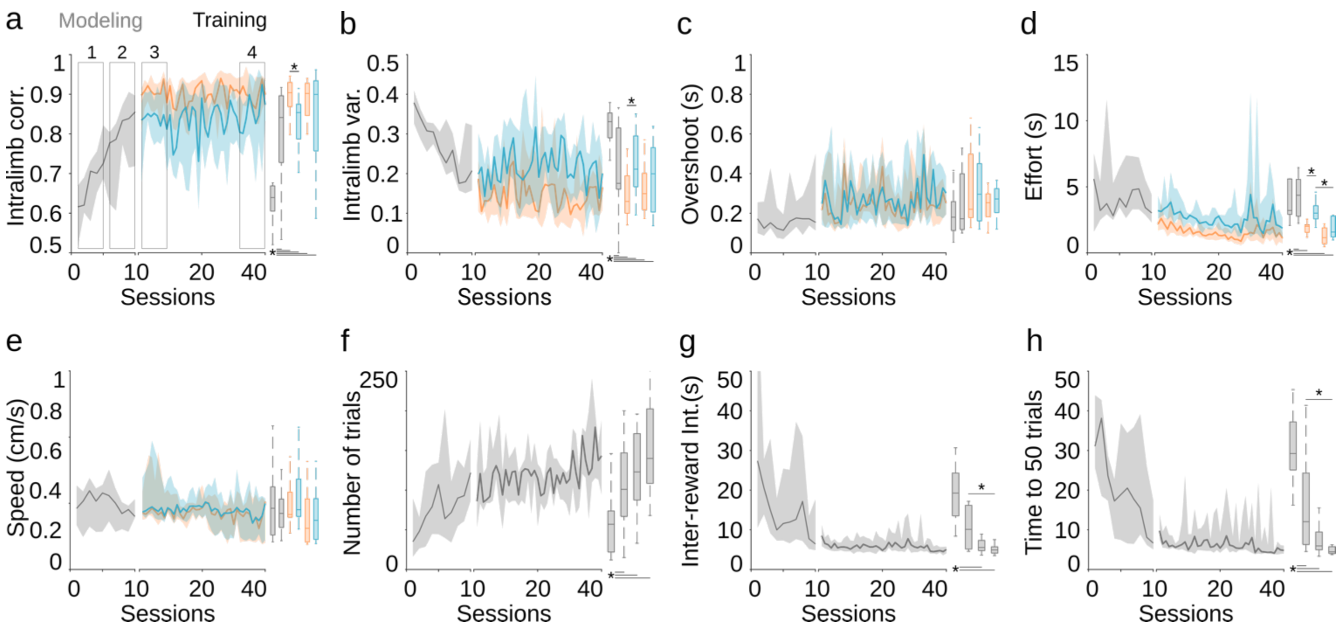

Supplementary Figure 6. Behavioral learning curves. (**a** to **h**) Learning curves (left panels) and boxplot comparisons for specific groups and sessions (right panels) including the modeling phase (gray) and the training phase in the two-interval version of the task (color coded) for the following variables: intralimb correlation **(a)**, variability (**b**), overshoot (**c**), effort (**d**), speed (**e**), number of trials (**f**), inter-reward interval (**g**), and time to reach the first 50 trials (**h**). Data is presented as median (solid line) ± 75th and 25th percentiles (shaded area). Boxplots indicate median and 75th and 25th percentiles for groups of five sessions at the four stages of the learning curves, indicated by the numbered rectangles in C. Statistical differences are indicated by asterisks and lines joining specific comparisons (Bonferroni post hoc test, P < 0.05). K-W values for C ($df = 5, X^2 = 31.76, p < 0.001$), D ($df = 5, X^2 = 25.9, p < 0.001$), E ($df = 5, X^2 = 5.15, p = 0.398$), F ($df = 5, X^2 = 30.48, p < 0.001$), G ($df = 5, X^2 = 4.43, p < 0.488$), H ($df = 3, X^2 = 16.41, p < 0.005$), I ($df = 3, X^2 = 21.73, p < 0.001$), J ($df = 3, X^2 = 26.87, p < 0.001$).

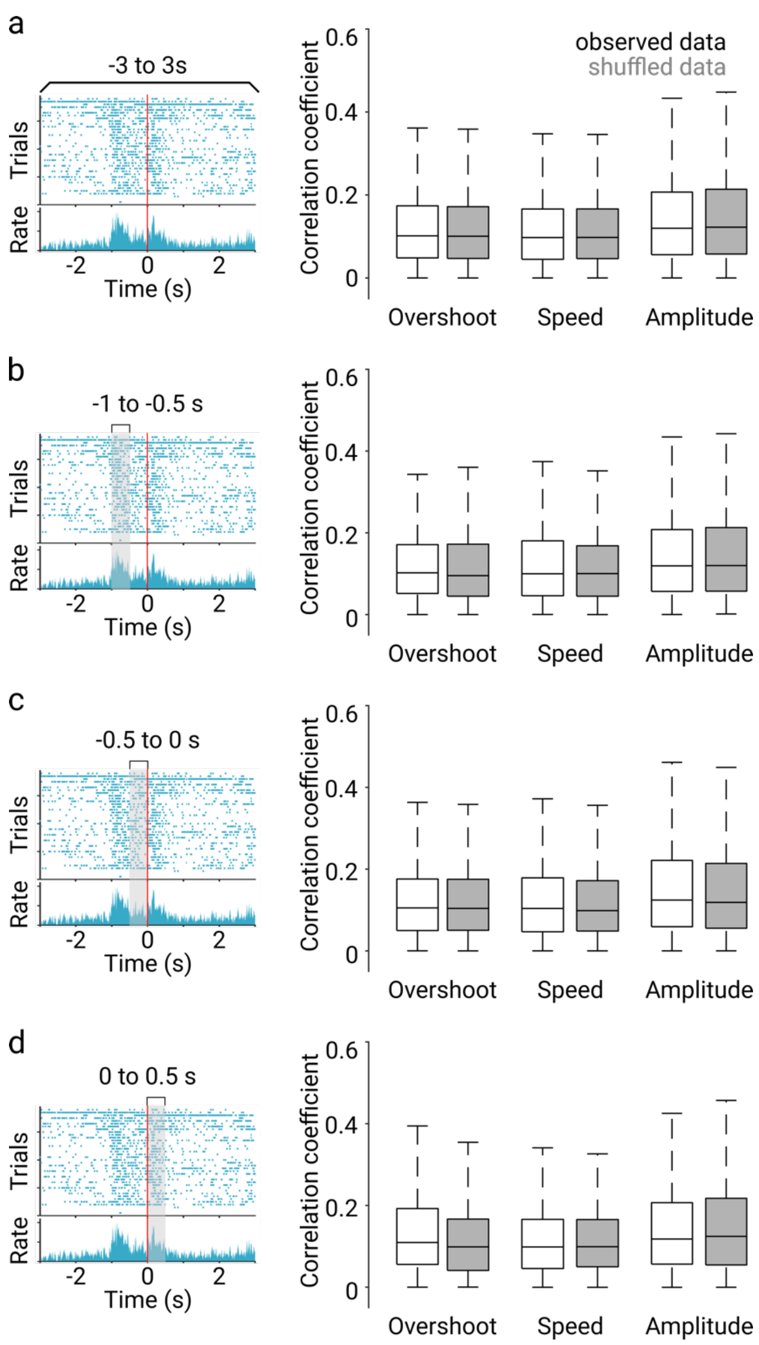

Supplementary Figure 7. Linear correlation analysis. Correlation coefficients (absolute values) for all recorded neurons and behavioral variables. Correlations were calculated between each variable and the average spiking activity from four periods indicated above the left rasters (shaded area displayed for reference). That is, the full -3 s to 3 s period before and after reward (**a**); -1 to -0.5 s before reward onset (**b**); -0.5 s before reward onset (**c**) and 0.5 s after (**d**) reward onset. The coefficients were obtained from the actual data (with boxplots) and from surrogated spike trains of the same data but with randomly shuffled spiking activity (gray boxplots). Boxplots indicate median and 75th and 25th percentiles. Statistical comparisons were performed by applying Kruskal-Wallis and Bonferroni post hoc tests.

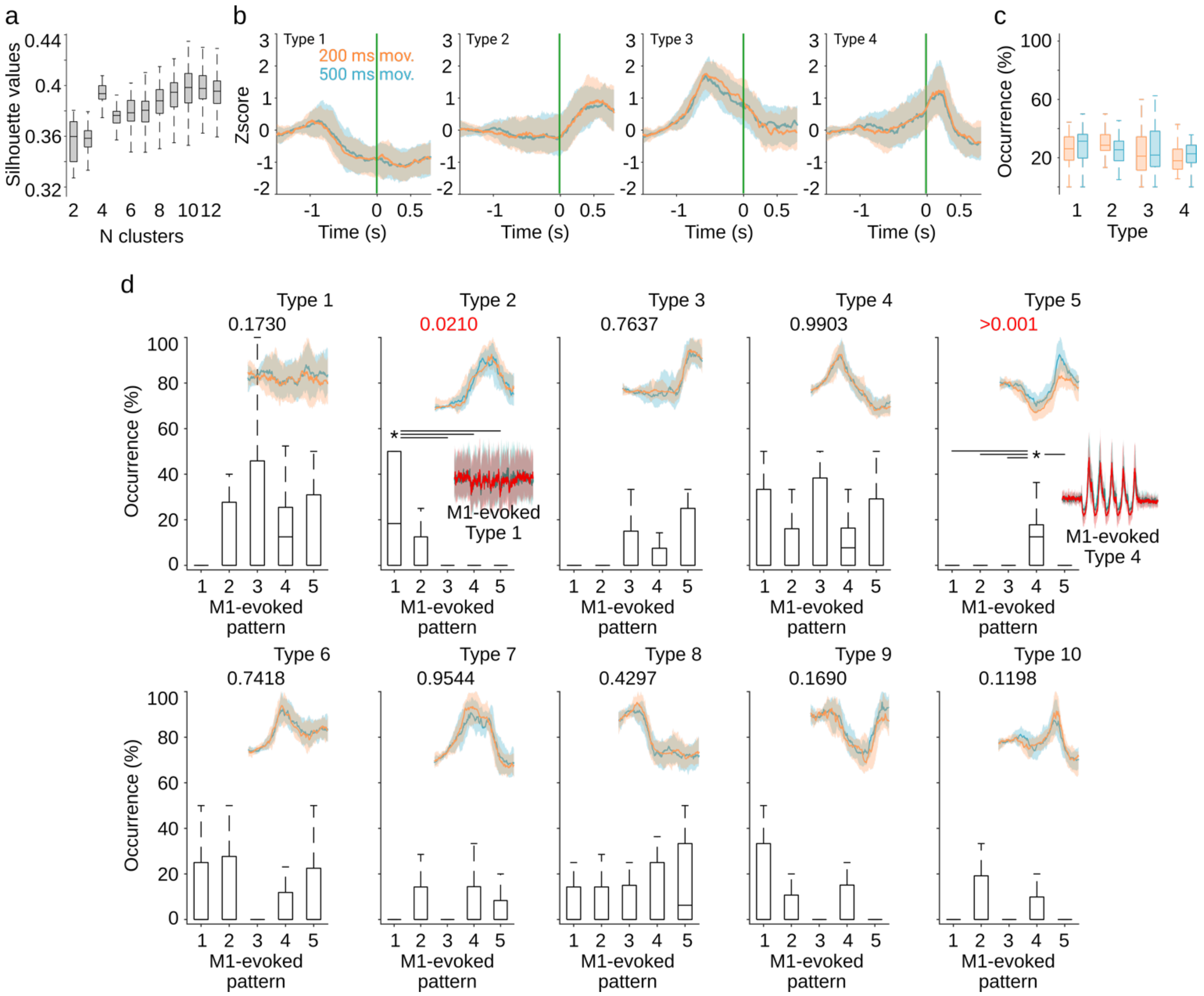

Supplementary Figure 8. Movement-related and M1-evoked patterns in VL/VM. a) Silhouette values for 1000 iterations in 2-12 k-means projections from the PCA on the peri-event histograms of the spiking activity during movement execution. **b**) Average behaviorally evoked peri-event histograms for cells classified as part of specific pattern clusters for the best four-cluster projection for the 200 ms (orange) and 500 ms (blue) conditions. **c**) Percentage of cells belonging to each pattern. Percentages were calculated using sessions with more than 10 cells simultaneously recorded. **d**) Percentage of cells belonging to each M1-evoked pattern (displayed in Figure 6B) as a function of each of the 10 behaviorally associated patterns (displayed as insets on each panel and taken from Figure 5E). Percentages were calculated using sessions with more than 10 cells simultaneously recorded. Boxplots indicate the median and 75th and 25th percentiles. Statistical differences are indicated by asterisks and lines joining specific comparisons (Kruskal-Wallis indicated above each panel and Bonferroni post hoc test, $P < 0.05$). For visual comparison, corresponding M1-evoked patterns from Figure 3 are depicted as insets in statistically significant panels.

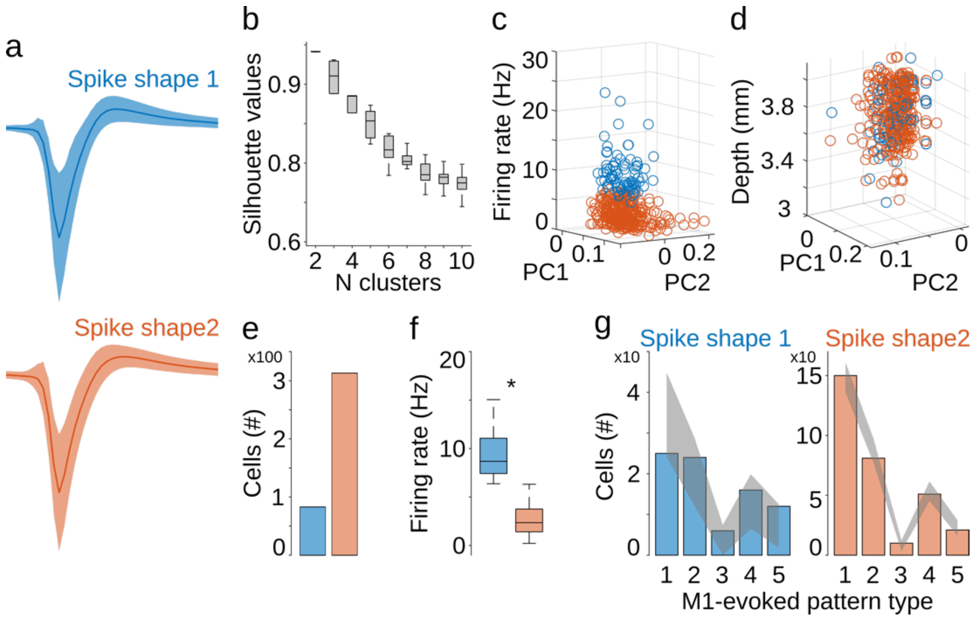

Supplementary Figure 9. Spike wave shape classification for VL/VM neurons. a) Average spike waves (solid line) for cells classified as part of two groups based on their spike shape, firing rate, and recording depth. **b**) Silhouette values for 1000 iterations in 2-10 k-means projections from the PCA on the average spike waves from all cells recorded during movement execution. **c, d**) 3D plots representing the best projection corresponding to two groups based on the Silhouette values from b (color coded). Principal components 1 and 2 were displayed together with firing rates (c) or recording depth (d). Number (**e**) and firing rates (**f**) for cells belonging each group. (**g**) Number of cells classified as spike shape (color coded) associated with a particular response pattern evoked by M1 stimulation (from Fig. 4b). Confidence intervals randomly generated from shuffled data neurons are depicted in gray shades for each pattern.

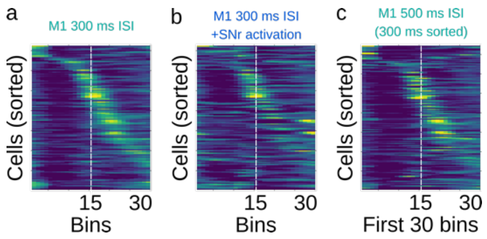

Supplementary Figure 10. SNr influence over VL/VM population temporal dynamics. M1-evoked sequential activation matrices (30 bins, 5 stimuli averaged) for 300 ms ISI trains with (**a**) and without (**b**) SNr-paired stimulation and for 500 ms inter-stimulus interval (ISI; **c**). The same neurons are presented in the three matrices and were sorted according to the moment of their highest firing rate during the 300 ms ISI (left).
