# Supplementary material for: Preconfigured cortico-thalamic neural dynamics constrain movement-associated thalamic activity
Source: Nat Commun. 2024 Nov 24;15:10185. doi: 10.1038/s41467-024-54742-9 (PMC11586408; doi:10.1038/s41467-024-54742-9)
Supplement: Supplementary file 2 — Reporting Summary [file 41467_2024_54742_MOESM2_ESM.pdf]

nature portfolio

Corresponding author(s): Pavel E. Rueda-Orozco

Last updated by author(s): Nov 6, 2024

Reporting Summary

Nature Portfolio wishes to improve the reproducibility of the work that we publish. This form provides structure for consistency and transparency in reporting. For further information on Nature Portfolio policies, see our Editorial Policies and the Editorial Policy Checklist.

Statistics

For all statistical analyses, confirm that the following items are present in the figure legend, table legend, main text, or Methods section.

n/a	Confirmed	
☐	☒	The exact sample size (*n*) for each experimental group/condition, given as a discrete number and unit of measurement
☐	☒	A statement on whether measurements were taken from distinct samples or whether the same sample was measured repeatedly
☐	☒	The statistical test(s) used AND whether they are one- or two-sided<br>*Only common tests should be described solely by name; describe more complex techniques in the Methods section.*
☒	☐	A description of all covariates tested
☐	☒	A description of any assumptions or corrections, such as tests of normality and adjustment for multiple comparisons
☐	☒	A full description of the statistical parameters including central tendency (e.g. means) or other basic estimates (e.g. regression coefficient) AND variation (e.g. standard deviation) or associated estimates of uncertainty (e.g. confidence intervals)
☐	☒	For null hypothesis testing, the test statistic (e.g. *F*, *t*, *r*) with confidence intervals, effect sizes, degrees of freedom and *P* value noted<br>*Give P values as exact values whenever suitable.*
☒	☐	For Bayesian analysis, information on the choice of priors and Markov chain Monte Carlo settings
☒	☐	For hierarchical and complex designs, identification of the appropriate level for tests and full reporting of outcomes
☒	☐	Estimates of effect sizes (e.g. Cohen's *d*, Pearson's *r*), indicating how they were calculated

Our web collection on statistics for biologists contains articles on many of the points above.

Software and code

Policy information about availability of computer code

Data collection	Electrophysiological data was acquired with RHX data acquisition software from Intan technologiesacquisition and processing (www.intantech.com). Data visualization and processing were performed from raw data using Neuroscope and NDManager (http://neurosuite.sourceforge.net). Spike sorting was performed semiautomatically using the clustering software KlustaKwik (http://klustakwik.sourceforge.net) and the graphical spike-sorting application Klusters (http://klusters.sourceforge.net).<br>All behavioral parameters and stimulation protocols were controlled and recorded with customized software programmed in LabView (version 2015).
Data analysis	All analyses were performed with customized routines based on already available Matlab (2018b, 2023b) functions.

For manuscripts utilizing custom algorithms or software that are central to the research but not yet described in published literature, software must be made available to editors and reviewers. We strongly encourage code deposition in a community repository (e.g. GitHub). See the Nature Portfolio guidelines for submitting code & software for further information.

Data

Policy information about availability of data

All manuscripts must include a data availability statement. This statement should provide the following information, where applicable:

- Accession codes, unique identifiers, or web links for publicly available datasets
- A description of any restrictions on data availability
- For clinical datasets or third party data, please ensure that the statement adheres to our policy

The datasets generated during the current study are available from the corresponding author on reasonable request

Research involving human participants, their data, or biological material

Policy information about studies with human participants or human data. See also policy information about sex, gender (identity/presentation), and sexual orientation and race, ethnicity and racism.

Reporting on sex and gender	*Use the terms sex (biological attribute) and gender (shaped by social and cultural circumstances) carefully in order to avoid confusing both terms. Indicate if findings apply to only one sex or gender; describe whether sex and gender were considered in study design; whether sex and/or gender was determined based on self-reporting or assigned and methods used. Provide in the source data disaggregated sex and gender data, where this information has been collected, and if consent has been obtained for sharing of individual-level data; provide overall numbers in this Reporting Summary. Please state if this information has not been collected. Report sex- and gender-based analyses where performed, justify reasons for lack of sex- and gender-based analysis.*
Reporting on race, ethnicity, or other socially relevant groupings	*Please specify the socially constructed or socially relevant categorization variable(s) used in your manuscript and explain why they were used. Please note that such variables should not be used as proxies for other socially constructed/relevant variables (for example, race or ethnicity should not be used as a proxy for socioeconomic status). Provide clear definitions of the relevant terms used, how they were provided (by the participants/respondents, the researchers, or third parties), and the method(s) used to classify people into the different categories (e.g. self-report, census or administrative data, social media data, etc.) Please provide details about how you controlled for confounding variables in your analyses.*
Population characteristics	*Describe the covariate-relevant population characteristics of the human research participants (e.g. age, genotypic information, past and current diagnosis and treatment categories). If you filled out the behavioural & social sciences study design questions and have nothing to add here, write "See above."*
Recruitment	*Describe how participants were recruited. Outline any potential self-selection bias or other biases that may be present and how these are likely to impact results.*
Ethics oversight	*Identify the organization(s) that approved the study protocol.*

Note that full information on the approval of the study protocol must also be provided in the manuscript.

Field-specific reporting

Please select the one below that is the best fit for your research. If you are not sure, read the appropriate sections before making your selection.

[x] Life sciences [ ] Behavioural & social sciences [ ] Ecological, evolutionary & environmental sciences

For a reference copy of the document with all sections, see nature.com/documents/nr-reporting-summary-flat.pdf

Life sciences study design

All studies must disclose on these points even when the disclosure is negative.

Sample size	No statistical method was used to determine the sample size. For all groups in electrophysiological recordings we report the total number of cells (always in the range of hundreds). Our sample sizes were similar than previous reports using similar recording and processing techniques (e.g. Rueda-Orozco et al, 2015; Hidalgo-Balbuena et al, 2019; Monteiro et al, 2023). For behavioral quantifications, the number of animals (dozens) was similar than in previous reports (e.g. Díaz-Hernández et al, 2018). Furthermore, the design of the task allowed us to generate hundreds to thousands of trials, increasing the statistical power.
Data exclusions	No data were excluded from the analysis
Replication	The electrophysiological signatures described in this work were robust and present in the great majority of the animals and under different experimental conditions. This was quantified and reported throughout the MS.
Randomization	In some comparisons we constructed random spike trains for the random distributions depicted in figures 2b and 5e. For each cell, each spike time from the spike trains obtained from the -1 to +4 s (or -4 to +4) s around the onset of the stimulation (or reward delivery) time windows, were circularly and randomly re-ordered by adding time in a range of + 1 to 5 (or + 1 to 8) s. For surrogated spontaneous activity depicted in

figure 6, we followed the same procedure but for the complete recorded spike trains and adding time in a range of a quarter of the length of the recording session.

Blinding

Data collection could not be performed blindly. Analysis was performed offline and Matlab codes were run in batch in all the data independently of the experimental condition.

Reporting for specific materials, systems and methods

We require information from authors about some types of materials, experimental systems and methods used in many studies. Here, indicate whether each material, system or method listed is relevant to your study. If you are not sure if a list item applies to your research, read the appropriate section before selecting a response.

Materials & experimental systems

n/a	Involved in the study
☒	☐ Antibodies
☒	☐ Eukaryotic cell lines
☒	☐ Palaeontology and archaeology
☐	☒ Animals and other organisms
☒	☐ Clinical data
☒	☐ Dual use research of concern
☒	☐ Plants

Methods

n/a	Involved in the study
☒	☐ ChIP-seq
☒	☐ Flow cytometry
☒	☐ MRI-based neuroimaging

Animals and other research organisms

Policy information about studies involving animals; ARRIVE guidelines recommended for reporting animal research, and Sex and Gender in Research

Laboratory animals

A total of 51 male mice were used in this study. From those, 40 animals were wild-type C57BL/6 and 11 were VGAT-Cre. Animals were 3- to 5-month-old male mice and were housed in individual acrylic boxes under temperature-controlled conditions with 12 hour light/dark cycles and free access to food and water.

Wild animals

The study did not involve wild animals

Reporting on sex

The study only used male subjects

Field-collected samples

No samples were collected from the field

Ethics oversight

All experiments were approved by the Animal Ethics Committee of the Institute of Neurobiology at the National Autonomous University of Mexico (UNAM) and conformed to the principles outlined in the Guide for the Care and Use of Laboratory Animals (National Institute of Health). Every precaution was taken to minimize suffering and the number of animals used in the experiments.

Note that full information on the approval of the study protocol must also be provided in the manuscript.

Plants

Seed stocks

Report on the source of all seed stocks or other plant material used. If applicable, state the seed stock centre and catalogue number. If plant specimens were collected from the field, describe the collection location, date and sampling procedures.

Novel plant genotypes

Describe the methods by which all novel plant genotypes were produced. This includes those generated by transgenic approaches, gene editing, chemical/radiation-based mutagenesis and hybridization. For transgenic lines, describe the transformation method, the number of independent lines analyzed and the generation upon which experiments were performed. For gene-edited lines, describe the editor used, the endogenous sequence targeted for editing, the targeting guide RNA sequence (if applicable) and how the editor was applied.

Authentication

Describe any authentication procedures for each seed stock used or novel genotype generated. Describe any experiments used to assess the effect of a mutation and, where applicable, how potential secondary effects (e.g. second site T-DNA insertions, mosiacism, off-target gene editing) were examined.
